# Felix Martin Oberländer (1851–1915) und sein Beitrag zur Wissenschafts- und Fachentwicklung von Urologie und Venerologie

**DOI:** 10.1007/s00120-022-01785-9

**Published:** 2022-03-30

**Authors:** Friedrich H. Moll

**Affiliations:** 1 Institut für Geschichte, Theorie und Ethik der Medizin, Heinrich-Heine-Universität Düsseldorf, Düsseldorf, Deutschland; 2 Museum, Bibliothek und Archiv zur Geschichte der Urologie Düsseldorf-Berlin, Düsseldorf-Berlin, Deutschland; 3 Deutsche Gesellschaft für Urologie e. V., Düsseldorf Berlin, Deutschland; 4 Urologische Klinik, Kliniken der Stadt Köln GmbH, Neufelder Straße 32, 51067 Köln, Deutschland

**Keywords:** Erinnerungskultur Urologie, Geschichte der Medizin, Wissenschaftsgeschichte, Medizin, Dresden, Culture of remembrance in urology, History of medicine, History of science, Medicine, Dresden

## Abstract

Während die Erinnerungskultur zu Maximilian Nitze innerhalb der deutschen Urologie gut entwickelt ist, bleibt das Wissen um Felix Martin Oberländer unscharf, obwohl er das *Zentralblatt für die Krankheiten der Harn- und Sexualorgane* herausgab, den Vorsitz bei der Gründung der alten Deutschen Gesellschaft für Urologie e. V. (DGU) im Jahre 1906 führte und später nach ihm ein Wissenschaftspreis der DGU benannt wurde.

## Abstract (en)

While the culture of remembrance of Maximilian Nitze, honorary member of the American Urological Association (AUA), has been cultivated, the contributions of Felix Martin Oberländer have been less noticed although he was an editor of the famous urologic journal *Zentralblatt für die Krankheiten der Harn- und Sexualorgane* (central journal for diseases of the urinary tract and sexual organs), was also honorary member of the AUA in 1902 and the main “founding father” of the German Society of Urology (DGU).

*Es ist fernerhin selbstverständlich, dass man wie bei anderen Instrumenten auch durch das Urethroskop, um erspriessliches zu leisten, erst sehen lernen muss* [1]

## Einleitung

Während in der Erinnerungskultur der deutschsprachigen Urologie [2, 3] und der Deutschen Gesellschaft für Urologie e. V. (DGU) der Name von Maximilian Nitze (1848–1906) deutlich verankert ist, obwohl gerade er nicht zum Begründer der Fachgesellschaft avanzierte, bleibt das erinnerungskulturelle Wissen um seinen Konassistenten am Dresdener Stadtkrankenhaus Friedrichstadt, Felix Martin Oberländer (08.01.1851–02.10.1915), der zum wesentlichen Promotor der Gründung einer deutschsprachigen urologischen Fachgesellschaft im Jahre 1906 avanzierte, gering. In der Regel bezogen sich die wenigen weiteren Untersuchungen [4, 5] auf die vorhandenen Nekrologe von Carl Posner (1854–1928), Hugo Lohnstein (1864–1918) sowie Oberländers Praxisassistenten Böhme [6–8]. Sie analysieren Oberländer zumeist in Zusammenschau mit Max Nitze in Fokussierung auf eine lokale Geschichte der Urologie sowie die Endoskopentwicklung [9, 10]. Seinen wichtigen Anteil an der Wissensentwicklung innerhalb des Faches und Konstituierung einer eigenständigen urologischen Krankheitslehre blieb unbekannt und unbeachtet. Gelegentlich wurde der Name noch in Zusammenhang mit der Therapie von Harnröhrenstrikturen zitiert [11, 12].

In der amerikanischen Erinnerungskultur ist die Position Oberländers mit dem gleichaltrigen und ein ähnliches wissenschaftliches Feld abdeckenden Ferdinand C. Valentine (1851–1909) besetzt, der selber ein Urethroskop angab, Gründungsmitglied der American Urological Association (AUA) war und nach dem ebenfalls Wissenschaftspreise in den USA benannt sind [13–15].

Doch bereits zu Lebzeiten war er von dem Medizinhistoriker Julius Pagel (1851–1912) in seinem Biographischen Lexikon aufgeführt worden, da er

… *durch schriftstellerische und wissenschaftlich-praktische Leistungen an dem Ausbau der Heilkunde in dem nun verflossenen, an Ergebnissen so reichen Jahrhunderte beteiligt…* war [16].

Auch im Biographischen Lexikon von Isidor Fischer (1868–1943 Bristol), Urban und Schwarzenberg 1933, ist der Name von Felix Martin Oberländer vermerkt [17].

Felix Martin Oberländer gehörte neben Maximilian Nitze (1848–1906) sowie Ernst Fürstenheim (1836–1904) und Paul Güterbrock (1844–1893) zur „älteren Urologenschule“ im deutschsprachigen Raum neben Victor von Ivanchich de Margita (1812–1892) und Robert Ultzmann (1842–1889) im Kaisertum Österreich.

In der Allgemeinen Deutschen Biographie (ADB) liegt eine Kurzinformation zu Oberländer vor [18].

Die folgende Untersuchung geht der Frage nach, welchen Anteil Felix Martin Oberländer an der Wissenschaftsentwicklung und Fachkonstitution der Urologie insbesondere im deutschsprachigen Raum besaß. Weiterhin möchte sie das teils weit verstreute Wissen um Oberländer zusammentragen und die Erinnerungskultur [19] zu Oberländer im Fachgebiet untersuchen.

## Zum Forschungsstand

Albrecht Scholz et al. (1940–2013; [4, 9, 10, 20–23]) hatten Felix Martin Oberländer im Rahmen der lokalen Dresdener Medizingeschichte und deren venerologischen Beitrag zur Medizingeschichte zwischen 1997–2009 mehrfach aspektiert, was zu einer Benennung eines Preises der Deutschen Gesellschaft für Urologie e. V. ab 1997 führte, ohne jedoch zunächst ein größeres erinnerungskulturelles Interesse zu wecken [24].^1^ 1999 erschien von der Medizinhistorikerin Susanne Zimmermann, Jena, ein Eintrag in der ADB [18]. Der Eintrag in dem Online-Lexikon „Wikipedia“ in deutscher und englischer Sprache fußt größtenteils auf den Dresdener Arbeiten [25]. In der sächsischen Biographie ist er ohne einen entsprechenden Artikel mit Namen aufgeführt [26].

## Biographische Skizze

Felix Martin Oberländer wurde 1851 als zweiter Sohn des Juristen und Geheimen Regierungsrates Martin Gotthard Oberländer (1801–1868; 2^,^^3^ [28–30]) der in Sachsen 1848–1849 als Innenminister im „Märzkabinett“ unter Karl Alexander Hermann Braun (1807–1868) fast ein Jahr berufen war und später weiterhin im sächsischen Innenministerium tätig bzw. Direktor der Brandversicherungskommission in Dresden war, in ein großbürgerliches, königstreues und wohlhabendes Elternhaus in Dresden geboren.

Schon der Großvater Martin Gotthart Oberländer^4^ (?–1852) war Mühlenbesitzer und Gerichtsschöffe in Langenbernsdorf gewesen.^5^ Seine Mutter war Charlotte Schumann, die bereits im Jahre 1832 den Sohn Richard (1832–1891), später Forschungsreisender und Kartograph, währen der Tätigkeit ihres Gatten in Zwickau, geboren hatte [31–34].

Felix Martin Oberländer besuchte die renommierte Dresdener Kreuzschule („schola crucis“) am Georgplatz und lege zu Ostern 1870 seine „Reifeprüfung“ ab, wobei er als Studienfach „Medizin“ angab [35].

**Figure 1:**
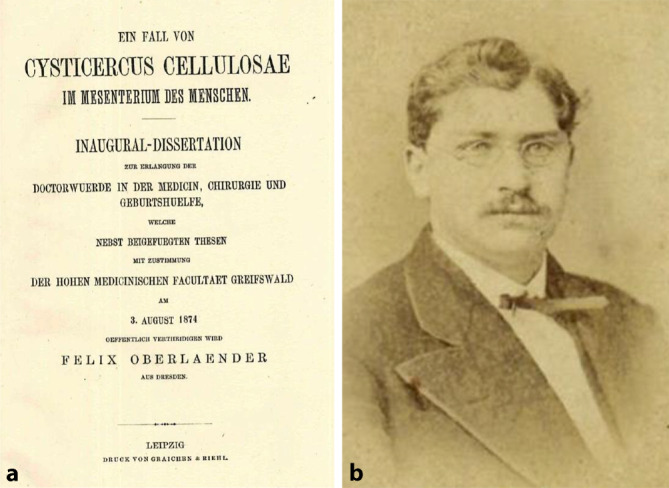

Danach studierte er Medizin in Leipzig (WS 1870/1871, SS 1871, WS 1871/1872) und Halle (SS 1872)^6^, wo er am 15.07.1872 das „Tentamen physicum“ ablegte.^7^ Danach wieder in Leipzig (WS 1872/1873)^8^, wechselte er im folgenden Semester nach Greifswald (SS 1873, WS 1873/1874 sowie SS 1874). Am 03.08.1874 promovierte er in Greifswald mit der Arbeit „Ein Fall von Cysticercus cellulosae im Mesenterium des Menschen“ [36]^9^, nachdem er am 15.03.1874 die erforderliche Staatsprüfung abgelegt hatte.^10^ Das Beiheften einer Abbildung bei einer Dissertation war zu dieser Zeit aufgrund der hohen Druckkosten von Abbildungen eher ungewöhnlich. Während seiner Leipziger Studienzeit war Oberländer Mitglied der pflichtschlagenden und farbentragenden Burschenschaft Dresdensia ([37, 38]; Abb. 1).

Danach trat er, typisch für die urologische Spezialarztausbildung am Ende des 19. Jahrhunderts, eine Studienreise zu den Weltzentren von Urologie und Venerodermatologie Paris, Wien [39] und Berlin^11^ [40] an. In Paris war das Hôpital Saint-Louis seit 1867 mit seiner bekannten Moulagensammlung ein internationaler Anziehungsort einer postgradualen venerologischen Ausbildung [41]. In Wien wurde dieses Spezialgebiet durch den Urologen und Syphidologen Josef Grünfeld (1840–1910; [42, 43]), der ab 1885 die „II. Abteilung für Syphilitische“ an der Wiener Allgemeinen Poliklinik leitete, schon während seiner Tätigkeit am Allgemeinen Krankenhaus unter Carl Ludwig Sigmund Ritter von Ilanor (1810–1883; [44]) prominent vertreten. In Berlin bestand um 1875 an der Charité („Neue Charité“, 200 Betten für Syphilitische) unter Richard Lewin (1820–1896) ein weiterer überregionaler Ausbildungsort [45]. Zwischen 1876–1878 war Felix Martin Oberländer zusammen mit dem wenig älteren Max Nitze (1848–1906) Assistenzarzt am Dresdener Stadtkrankenhaus Friedrichstadt, am Ende des 19. Jahrhunderts die zweitgrößte „Krankenanstalt“ in Sachsen unter Julius Otto Martini (1829–1909) in der Zweiten Äußeren Abteilung [21, 22, 46].^12^

Hier konnte er die Arbeiten von Nitze zur Beleuchtung von Körperhöhlen verfolgen sowie die erste Demonstration des Zystoskops an der Leiche am 02.10.1877 vor dem Königlich Sächsischen Landesmedizinalkollegium, als höchster medizinische Behörde des Königreichs Sachsen [48, 49]. Entsprechend seines eigenen akademischen venerourologischen Schwerpunkts, der Therapie urethraler Erkrankungen, war Felix Martin Oberländer besonders am Urethroskop interessiert. Somit konnte er wissenschaftlich den auf seiner Priorität beharrenden Max Nitze bei der Wahl seines eigenen Instrumenteninteresses ausweichen und auch späterhin damit ein eigenständiges Forschungsfeld in seiner eigenen Praxis und Privatklinik aufbauen.

Arthur Kollmann stellte hierzu im Jahre 1930 als Zeitzeuge fest:

Im Gegensatz zu diesen beiden Arten der Beleuchtung von außen verlegte Nitze (1879) die Lichtquelle in das Innere des Tubus, eine Methode, die von Oberländer für die Harnröhre ausgebaut wurde. Mit Hilfe dieses Instruments schuf Oberländer den Grundstock der Urethroskopie. Das Oberländer-Instrument erhielt später eine für die Handhabung günstige Verbesserung dadurch, daß es Valentine gelang, eine genügend kleine elektrische Glühbirne anfertigen zu lassen, die den lichtspendenden, glühenden Platindraht des Oberländer-Instruments ersetzte und dadurch auch die Wasserspülung überflüssig machte, – ein Gedanke übrigens, der von Oberländer und Kollmann schon vorher konzipiert und ausprobiert wurde, der aber bei dem damaligen Stande der Technik in der Glühlampenfabrikation Deutschlands ein so unvollkommenes Resultat ergab, daß seine Verwirklichung sich darauf beschränken mußte, ein interessantes Stück in Oberländers und Kollmanns Museum urethroskopischer Instrumente zu bleiben …[50]

**Figure 2:**
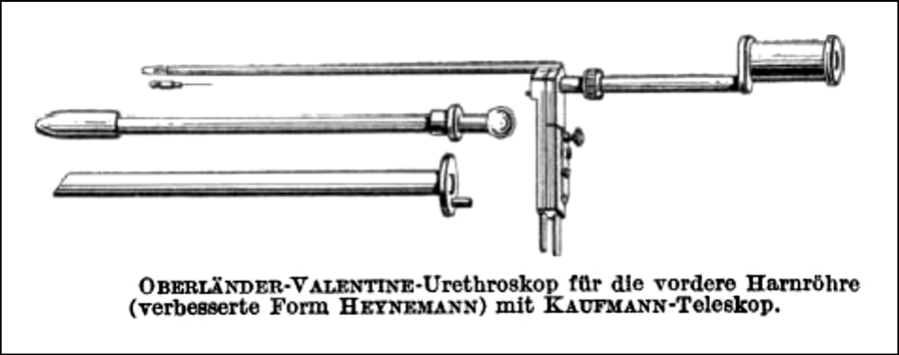

… Das von Oberländer ausgebaute Urethroskop nach dem Prinzip der Innenbeleuchtung von Nitze ist heute ausschließlich mit der kleinen elektrischen Glühbirne Valentines ausgerüstet. In dieser Form ist es jetzt das gebräuchlichste Instrument für die „trockene“ Endoskopie der vorderen Harnröhre … ([50]; Abb. 2)

Das Dresdener Adressbuch weist Oberländer für das Jahr 1878 noch als Assistenzarzt am Stadtkrankenhaus aus [51]. In der nächsten Ausgabe von 1880 wird er in eigener Praxis Schloßstraße 27 als praktischer Arzt aufgeführt, ab 1882 schon in der gut frequentierten Prager Straße 9, II. Etage, später ab 1884 unter Nr. 7 auf der patientenfreundlichen und repräsentativen I. Etage und mit weiteren Sprechstundenzeiten in der Poliklinik Zeughofplatz 3. In der Architektur des 19. Jahrhunderts war die erste Etage als „die belle etage“ im Vorderhaus besonders repräsentativ, mit aufwendigerem Stuck bei höheren Räumen ausgestattet, besaß beim Gasglühlicht mehr Brennstellen und war „Herrschaften und Beamten“ als sozialem Distinktionsmerkmal vorbehalten. Das Proletariat musste im Anbau oder Hinterhaus leben und mit einem Abort auf der „Versenkgrube“ im Hof vorlieb nehmen, während zumeist englische „Spülklosetts“ ab 1870 mit Einführung einer Schwemmkanalisation im Vorderhaus verbaut wurden. Die Prager Straße, die den Dresdener Hauptbahnhof (Böhmischer Bahnhof) mit dem Altmarkt verbindet, war bereits im 19. Jahrhundert die bedeutendste Geschäftsstraße der Residenzstadt. Seit dem Jahre 1889, nach 10 Jahren in freier Praxis, wurde im Adressbuch der Vermerk „prakt. Arzt“ durch „Spezialarzt für Krankheiten der Harnorgane“ ersetzt. Dies lässt darauf schließen, dass auch mit dieser Spezialisierung ein auskömmliches Einkommen gegeben war und sich Oberländer einen besonderen Ruf beim Publikum erworben hatte. Zu dieser Zeit war bei Ärzten in freier Praxis in der Regel Wohn‑/Praxisadresse identisch.

Im Jahre 1914 wird die Praxis unter Christianstraße 28, 1. Etage, aufgeführt und Oberländers Privatwohnung separat mit Dresden-Blasewitz, Residenzstraße 40 angegeben [52]. Blasewitz zählt zu den historischen Villenvororten Dresdens am linken Elbufer. Durch den Zuzug von Fabrikanten, hohen Beamten und Offizieren war Blasewitz die Gemeinde mit dem höchsten Steueraufkommen im Königreich Sachsen. Der Stadtteil war und ist überregional bekannt durch das sog. „Blaue Wunder“, dem inoffiziellen Namen der Loschwitzer Brücke, eine der über die Elbe führenden Brücken. Sie verbindet die Stadtteile Blasewitz am linken und Loschwitz am rechten Ufer der Elbe miteinander und galt als technisches Meisterwerk ihrer Zeit.

In späteren Jahren, ab 1909, führte Felix Martin Oberländer seine Praxis zusammen mit Fritz Boehme (1875–1932)^13^. Im Jahre 1932 wurde die Praxis von Max Funfack (1895–1972; [53]) übernommen, der bereits 1925 als Sozius eingestiegen war und diese bis 1968 weiterführte.

Zwischen 1880–1893 war Oberländer als Polizeiarzt tätig [4]. In dieser Eigenschaft war er für die „14-tägige Untersuchung von 600 Prostituierten und Vagantinnen“ zuständig und hierdurch war ihm „für die Beobachtung gonorrhoisch erkrankter Vaginalschleimhäute reichlich Gelegenheit geboten“, analog seinem Kollegen und Koautor Arthur Kollmann (1858–1941) in Leipzig. Seine Erfahrungen flossen in eine Publikationen ein [54].

Oberländer war als Orchideenzüchter Mitglied der Gesellschaft „Flora – Sächsische Gesellschaft für Botanik und Gartenbau“ in Dresden.^14^

Aus seiner Ehe ging ein Sohn hervor.

**Figure 3:**
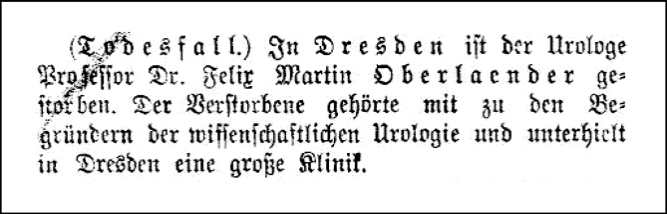

Felix Martin Oberländer verstarb am 02.10.1915^15^ [6–8, 55] und wurde in Dresden auf dem Johannisfriedhof^16^ beigesetzt. In Nachrufen, die auch in allgemeinen Zeitungen und Handbüchern [56] erschienen, [57, 58] wurde besonders seine umfangreiche klinische Tätigkeit herausgestellt sowie sein Einfluss auf das sich entwickelnde Fach hervorgehoben (Abb. 3).

Da er sich immer wieder in seinen Texten an seinen Konassistenten Max Nitze zurückband, konnte er auf ein gemeinsames Ziel seines wissenschaftlichen Denkkollektivs hinarbeiten [59].

## Oberländers Œuvre in wissenschaftlichem und fachpolitischen Bereich

**Figure 4:**
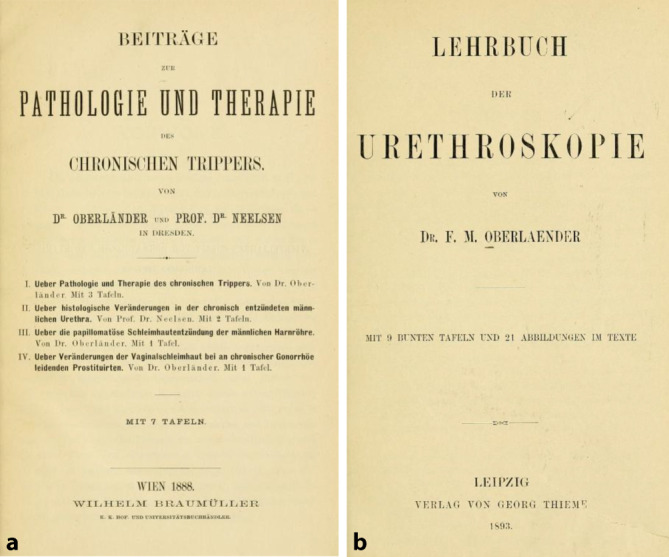

Felix Martin Oberländer (Abb. 4) entfaltete ein reges wissenschaftliches und berufspolitisches Wirken gleichzeitig zu seiner freien Praxistätigkeit, wie es bei Urologen dieser Generation nicht unüblich war. Er publizierte Einzelbeiträge in renommierten Zeitschriften [1, 54, 60–73], wie der „Vierteljahresschrift für Dermatologie und Syphilis“, seriellen Publikationen und Handbüchern ([64, 74–79]; Abb. 4).

Da es in den 1880er-Jahren des 19. Jahrhunderts für die Urologie noch keinen publizistisch definierten Ort gab, erschienen Beiträge zum Fachgebiet in chirurgischen, venerologischen, internistischen oder allgemeinmedizinischen (*Berliner* bzw. *Münchener* oder *Wiener medizinische/klinische Wochenschrift*) Publikationsorganen. Dies war sicherlich ein Grund, dass Oberländer ab 1893 Mitherausgeber des neuen meinungsbildenden *Centralblatt für die Physiologie und Pathologie der Erkrankungen des Harn- und Sexualapparates* (Hrsg.: Oberländer-Nitze) wurde, das nunmehr im deutschsprachigen Raum das sich entwickelnde Fachgebiet fokussieren konnte. Es wurde 1907 nach Vereinigung mit den *Monatsberichten für Urologie* (Hrsg. Casper-Lohnstein) zur renommierten *Zeitschrift für Urologie*, die bis 1990 bestand und mit der Wiedervereinigung aus verlagstaktischen Gründen unterging.

**Figure 5:**
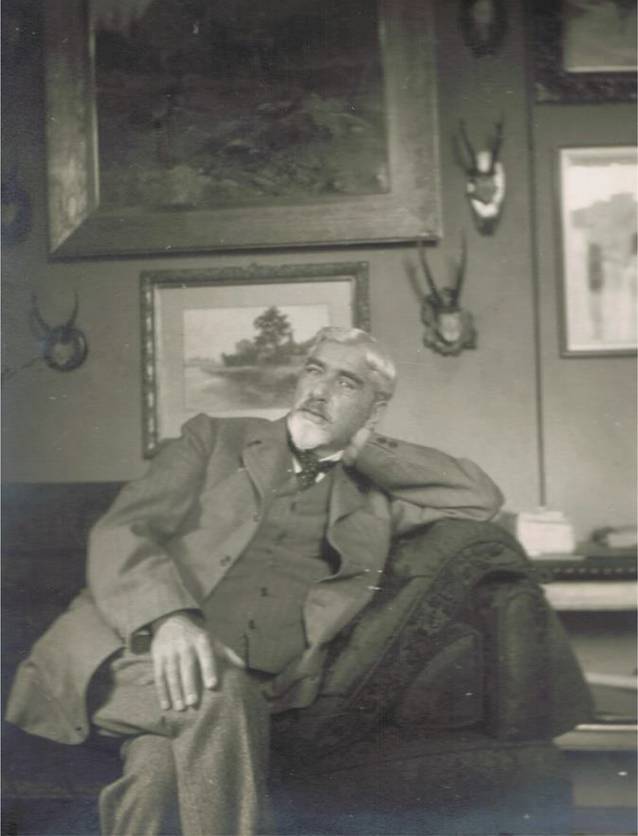

Sein Beitrag „Die chronischen Erkrankungen der männlichen Harnröhre“ in dem von ihm selber mit Wilhlem Zuelzer (1834–1893; [80]^17^), Berlin, 1894 herausgegebenen ersten deutschsprachigen Handbuchs zur Urologie „Klinisches Handbuch der Harn- und Sexualorgane“ bei dem renommierten Verlagshaus FCW Vogel, veranschaulicht seine arrivierte Stellung im Fachgebiet. Die Zeitgenossen lobten in Buchrezensionen den Autor (Abb. 5).

*…welcher der ihm gestellten Aufgabe mit grossem Geschick gerecht geworden ist* [81]

Im Jahre 1911 erfolgte die Gründung des „Vereins Dresdener Dermatologen und Urologen“ [22, 82] durch den Dermatologen Johannes Werther (1865–1936), dessen Fortbildungsabende überregionale Bekanntheit besaßen. Im Jahr 1926 wurde die Verbindungen des Vereins zum Gebiet der Urologie gelöst [5]. Das hatte sicherlich auch einen Grund darin, dass 1924 für das Deutsche Reich die Facharztfrage auf dem Bremer Ärztetag geregelt worden war und die Urologie als eigenständige Disziplin verankert wurde.

In die Eponymik der Urologie ging Felix Martin Oberländer mit mehreren Instrumenten nachhaltig ein, die bis zur Einführung der antibiotischen Therapie der Gonorrhö international in Gebrauch waren.

**Figure 6:**
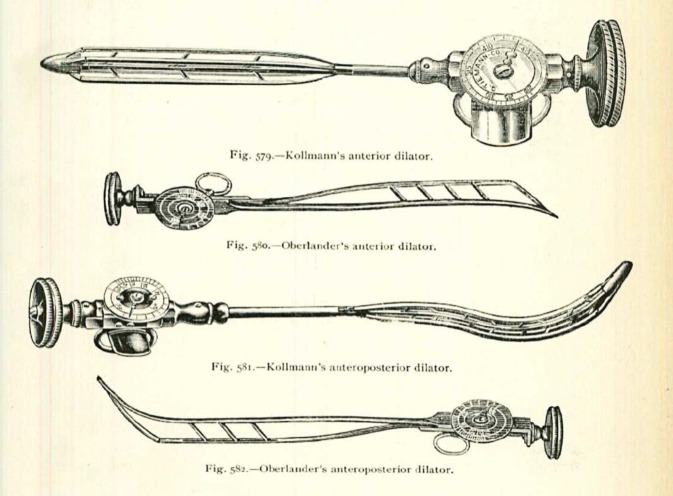

Sein Beitrag zur „trockenen“ endoskopischen Therapie von Harnröhrenstrikturen durch sein Urethroskop gilt bis heute als richtungsweisend ([83]; Abb. 6, 7 und 8).

**Figure 7:**
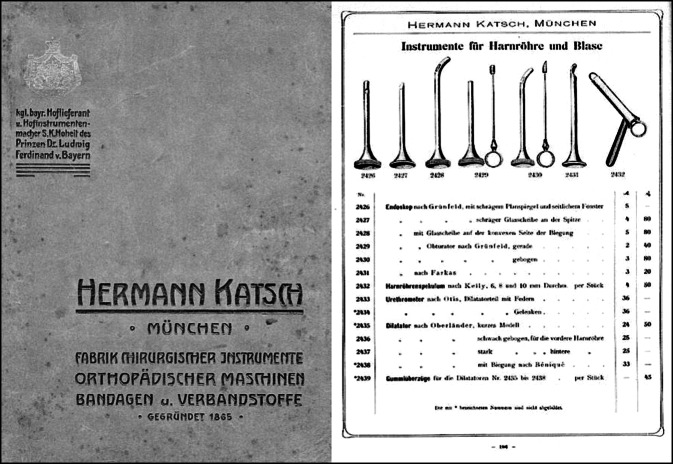

**Figure 8:**
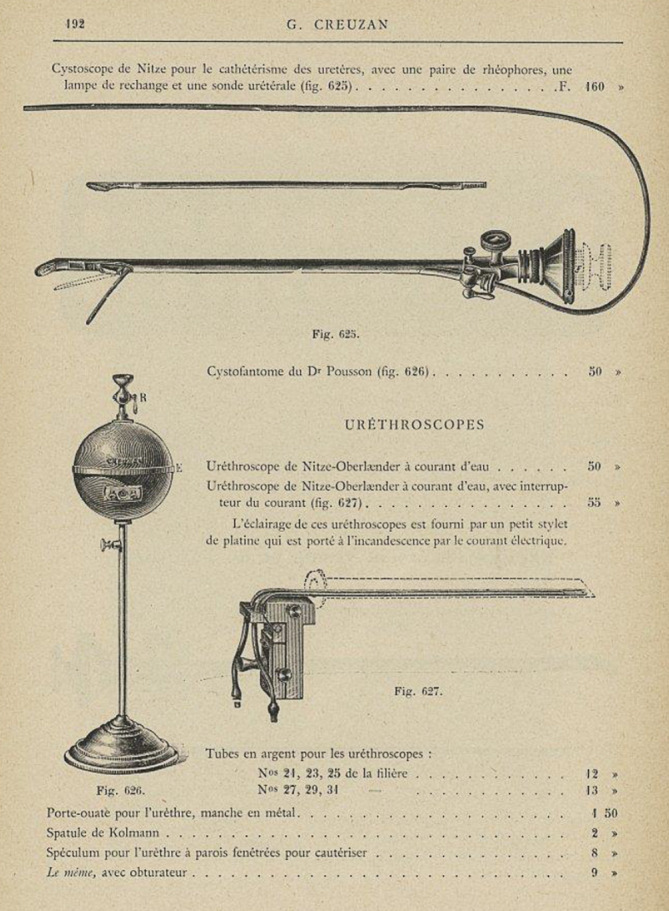

## Gründung der Deutschen Gesellschaft für Urologie e. V.

Obwohl zur eigentlichen Konstituierung der Deutschen Gesellschaft für Urologie e. V. originale Quellen weithin fehlen, kann der Gründungsprozess anhand von Sekundärquellen und Egodokumenten heute nachvollzogen werden [84]. In den Netzwerken der aktiven Forscher spielte Felix Martin Oberländer hierbei eine wichtige, herausragende und prägende Rolle, nicht nur als Vertreter der sächsischen Schule, die neben den Berliner Urologen maßgeblich an der Gründung der Fachgesellschaft beteiligt waren. Er gehörte neben Arthur Kollmann aus Leipzig zu denjenigen, die sich auf der Stuttgarter Naturforscherversammlung 1906 nach dem Tode des „charakterlich schwierigen“ Maximilian Nitze trafen, der eine Gründung der Gesellschaft wohl spätestens seit 1896 behindert hatte, um nun die Aktivitäten, die schon 1896 eingeleitet worden waren, aktiv persönlich voranzutreiben. Aufgrund seines Alters, seiner lange bekannten klinischen Exzellenz mit Versorgung eines überregionalen Patientenguts und ausgewiesener wissenschaftlichen Reputation in Einzelpublikationen und Handbuchbeiträgen, Oberländer war zu diesem Zeitpunkt 55 Jahre alt, war er neben Carl Posner, 52 Jahre alt, und dem jüngeren Leopold Casper, 48 Jahre alt, besonders hierzu prädestiniert. Er leitete daher als „Alterspräsident“ die konstituierende Sitzung der neuen medizinischen Fachgesellschaft [85].

Auf der Sitzung stellte Oberländer in der Überlieferung von Carl Posner fest:

*Wir haben uns allmählich und sicher, unabhängig von jeder anderen Disziplin, auf eigene Füße und fest zu stellen gewußt, und diese neue Stellung soll heute durch unsere Gründung nach außen hin dokumentiert werden *[6].

Dieser Satz unterstreicht deutlich das Selbstbewusstsein und das eigene Erleben des Zeitzeugen Oberländer für eine eigenständige urologische Fachentwicklung und versucht gleichzeitig dem bereits existierenden Narrativ, die Urologie sei eine Untergliederung der Chirurgie – eine Bindestrichchirurgie wie es Friedrich Voelcker (1872–1955), der sich selber wissenschaftlich hauptsächlich mit der Urochirurgie beschäftigte und hier eine eigentümliche Sicht auf sein eigenes Tun zeigt, auf dem Chirurgenkongress 1932 formulierte, entgegenzutreten [86].

## Fazit

Neben Arthur Kollmann in Leipzig gehörte Felix Martin Oberländer zu den Vertretern der sächsischen Urologenschule, die neben der älteren Berliner Schule um Max Nitze, Paul Güterbrock (1844–1893) und Ernst Fürstenheim (1836–1904) die Fachspezialisierung in der Regel in eigener, niedergelassener Praxis, teils an Hochschulen assoziiert, vehement vorantrieben und durch ihre jeweiligen lokalen, aber auch internationalen Netzwerke zu den Nachbardisziplinen wie Chirurgie, Venerodermatologie und auch Frauenheilkunde maßgeblich prägten. Aus diesem Grund wurden Vertretern aus beiden Gruppen, der Berliner und Dresdener, bei der Gründung der American Urological Association (AUA) im Jahre 1902 als Ehrenmitglieder aus dem Deutschen Reich berücksichtigt. Obwohl Oberländers Name für Instrumente wie Urethraldilatatoren oder Urethroskopen eponymbildend wurde, ist er in der Erinnerungskultur des Faches, nicht der Fachgesellschaft, weitgehend in Vergessenheit geraten. Dies hat sicherlich auch einen Grund darin, dass sein Forschungsfeld, die Behandlung gonorrhoischer Harnröhrenaffektionen seit Einführung der Antibiotika nach dem Zweiten Weltkrieg an Bedeutung verloren hat.

## Footnotes

1 Der Preis wird von der Fa. Apogepha, Dresden, unterstützt; Verdienste in der Fort- und Weiterbildung auf dem Gebiet der Urologie. Preisträger: 1997 H. Madersbacher; 1998 K. Naber; 1999 J. Altwein; 2000 W. Weidner; 2001 R. H. Ringert; 2002 M. Stöhrer; 2003 A. Hesse; 2004 H. Heidler; 2005 R. Harzmann, 2006 P. Fornara; 2007 R. Beetz; 2008 K. Dreikorn; 2009 H, J. Berberich; 2010 J. Rassweiler; 2011 A Semjonow; 2012 K. Höfner; 2013 H. Leyh; 2014 D. Schultz-Lampel; 2015 H. J. Piechota; 2016 S. Michel; 2017 U. Otto; 2018 T. Knoll, 2019 S. Kliesch; 2020 F. Moll; 2021 T. Kälble, St. Roth.

2 Dekret an die Stände: Die Vorlegung der Entwürfe zu den provisorischen Gesetzen, die Wahl der Landtagsabgeordneten und einige Abänderungen in der Verfassungsurkunde betreffend: eingegangen bei der II. Kammer am 4. September 1848 Oberländer, Martin Gotthart. – [Dresden], 1848.

3 Stadtgerichtsaktuar und Vizestadtrichter in Zwickau, 1835 bis 1848 Erster Stadtrat und Vizebürgermeister in Zwickau, ab 1842 Quelle [27].

4 auch andere Schreibungen wie Gotthard in Gebrauch.

5 Oberländer, Martin Gotthart (1801–1868) (07.05.1801, Langenbernsdorf–16.05.1868, Dresden) in [27].

6 UAHW, Rep. 39, Nr. 41.

7 Lebenslauf Vita aus Dissertation o. S. Universitätsarchiv Greifswald.

8 UAL_Rep_01_16_07_C_034_Bd_1_07 02 1873.

9 Schreiben Universitätsarchiv Greifswald Archivarin Frau Marike Zenke vom 29.07.2020 „Zum *Promotion*svorgang an der Medizinischen Fakultät gibt es eine Sammelakte, die die Unterlagen mehrerer Promovenden umfasst. Zu Felix Oberländer, der am 3. August 1874 hier promovierte, enthält die Akte folgende Dokumente: Promotionsurkunde (großformatig, gefaltet); Dissertationsschrift mit Vita; Anmeldung, Gesuch und Protokoll zum Examen rigorosum (zusammen mit zwei weiteren Kandidaten); handschriftliche Vita; handschriftliche Abhandlung zum Thema: Anatomie und Physiologie der Nieren von menschlichem Körper.“

10 Lt. Matrikeleintragung Nr. 209 vom Sommersemester 1873 hat er am 30. April 1873 sein Studium der Medizin hier in Greifswald aufgenommen und ist Michaelis 1874 von der Universität abgegangen (Matrikel Bd VIII), 3. August 1874 Promotion. Schreiben Universitätsarchiv Halle 27.06.2020.

11 Da Max Funfak aus Dresden stammte, kann eine gewisse Sicherheit bestehen, dass dieser Studienort, der in anderen Quellen nicht genannt war, noch in der 1. Hälfte des 20. Jahrhunderts für Oberländer in Erinnerung war.

12 Die Station war am Stadtkrankenhaus Dresden-Friedrichstadt ab 1866 der Chirurgie zugeordnet und erhielt 1874 einen eigenständigen Status. Während sich die Dermatologie aus der Medizinischen Klinik zu Beginn des 19. Jahrhunderts entwickelte, besaß die Venerologie über die operative Spaltung der Bubonen und der Harnröhren Eingriffe bei Tripper eine Verbindung zur operativen Medizin. [47].

13 Fritz Böhme war Angehöriger der Corps Guestphalia Jena und Lusatia Leipzig. Kösener Corpslisten 1960, 70/317; 3/694.

14 Ecce 1915 der Crucianer, 1915, S. 11–12., „[Er] widmete sich mit besonderem Eifer und großem Erfolge der Pflanzenzucht, namentlich der Zucht von [Orchideen], wodurch er sich einen weit über das Vaterland hinausreichenden Ruf erwarb. Er veranstaltete zu wohltätigen Zwecken Ausstellungen in seinem Gewächshäusern und erhielt für seine Beschickung von Ausstellungen goldene und silberne Medaillen …“

15 Sterbebuch(register) des Standesamtes Blasewitz, jetzt Dresden, vom 2. Oktober 1915, Urkunde Nr. 84.

16 Johannisfriedhof in Dresden-Tolkewitz, Feld 4, Bogen, 045.

17 Zuelzer las an der Berliner Friedrich-Wilhelms-Universität „Krankheiten der Harn- und Sexualorgane“.
